# Role of the kidneys in acid-base regulation and ammonia excretion in freshwater and seawater fish: implications for nephrocalcinosis

**DOI:** 10.3389/fphys.2023.1226068

**Published:** 2023-06-29

**Authors:** Marius Takvam, Chris M. Wood, H. Kryvi, Tom O. Nilsen

**Affiliations:** ^1^ Department of Biological Sciences, University of Bergen, Bergen, Norway; ^2^ Department of Zoology, University of British Columbia, Vancouver, BC, Canada

**Keywords:** acid-base regulation, kidney stones, patophysiology, transporters and channels, nephron tubule, aquaculture, water quality (WQ), fish

## Abstract

Maintaining normal pH levels in the body fluids is essential for homeostasis and represents one of the most tightly regulated physiological processes among vertebrates. Fish are generally ammoniotelic and inhabit diverse aquatic environments that present many respiratory, acidifying, alkalinizing, ionic and osmotic stressors to which they are able to adapt. They have evolved flexible strategies for the regulation of acid-base equivalents (H^+^, NH_4_^+^, OH^−^ and HCO_3_^−^), ammonia and phosphate to cope with these stressors. The gills are the main regulatory organ, while the kidneys play an important, often overlooked accessory role in acid-base regulation. Here we outline the kidneys role in regulation of acid-base equivalents and two of the key ‘urinary buffers’, ammonia and phosphate, by integrating known aspects of renal physiology with recent advances in the molecular and cellular physiology of membrane transport systems in the teleost kidneys. The renal transporters (NHE3, NBC1, AE1, SLC26A6) and enzymes (V-type H^+^ATPase, CAc, CA IV, ammoniagenic enzymes) involved in H^+^ secretion, bicarbonate reabsorption, and the net excretion of acidic and basic equivalents, ammonia, and inorganic phosphate are addressed. The role of sodium-phosphate cotransporter (Slc34a2b) and rhesus (Rh) glycoproteins (ammonia channels) in conjunction with apical V-type H^+^ ATPase and NHE3 exchangers in these processes are also explored. Nephrocalcinosis is an inflammation-like disorder due to the precipitation of calcareous material in the kidneys, and is listed as one of the most prevalent pathologies in land-based production of salmonids in recirculating aquaculture systems. The causative links underlying the pathogenesis and etiology of nephrocalcinosis in teleosts is speculative at best, but acid-base perturbation is probably a central pathophysiological cause. Relevant risk factors associated with nephrocalcinosis are hypercapnia and hyperoxia in the culture water. These raise internal CO_2_ levels in the fish, triggering complex branchial and renal acid-base compensations which may promote formation of kidney stones. However, increased salt loads through the rearing water and the feed may increase the prevalence of nephrocalcinosis. An increased understanding of the kidneys role in acid-base and ion regulation and how this relates to renal diseases such as nephrocalcinosis will have applied relevance for the biologist and aquaculturist alike.

## Introduction

Acid-base regulation is vital for all vertebrates to maintain homeostasis of intra- and extracellular pH. Maintenance of normal pH levels is essential for homeostasis in many physiological systems and one of the most tightly regulated physiological processes among vertebrates. In contrast to terrestrial animals, fish live in aquatic environments, and must thus utilize different strategies to regulate acid-base equivalents (H^+^, NH_4_^+^, OH^−^ and HCO_3_^−^), ammonia and phosphate. Recent reviews (Tresguerres et al., 2020; Zimmer and Perry, 2022) provide a useful overview of acid-base regulation and ammonia excretion in fish, and since the two processes are intimately related, they will be considered together. For both processes, there is general accord that the gills constitute the major regulatory site, while the kidneys play an important accessory role. In terms of acid-base balance, excretion of H^+^ or NH_4_^+^ both represent acidic equivalent excretion, whereas excretion of OH^−^ or HCO_3_^−^ both represent basic equivalent excretion (Hills, 1973; Davenport, 1974). Disturbances of acidic or basic equivalents are traditionally termed “metabolic disturbances”. The respiratory gases CO_2_ and NH_3_ certainly affect the pH of the body fluids (CO_2_ is acidifying, whereas NH_3_ is alkalinizing), but in classic acid-base terms, these are not considered to be acidic or basic equivalents, but rather “respiratory factors” that affect acid-base homeostasis. As the typical pH of fish body fluids (7.2—8.2) is far above the pK of the CO_2_ - HCO_3_^−^ interconversion, and far below the pK of the NH_4_^+^ - NH_3_ interconversion, HCO_3_^−^ and NH_4_^+^ are the species that greatly predominate - generally >95% in terms of concentration in the body fluids (Randall and Wright, 1989). However, it is the uncharged gases (CO_2_ and NH_3_) that move easily across cell membranes, whereas the movements of the charged ions HCO_3_^−^ and NH_4_^+^ (basic and acidic equivalents respectively) must be facilitated by transporters or channels.

Nephrocalcinosis is an inflammation-like disorder due to precipitation of calcareous material in the kidneys. In salmonid aquaculture, the prevalence of nephrocalcinosis appears to increase, and is primarily considered a problem in land-based systems (i.e., ‘freshwater and brackish water’) but is also observed in the seawater phase (Klykken et al., 2022a). The causative links underlying the pathogenesis and etiology of nephrocalcinosis in teleosts are speculative at best, but acid-base perturbations is assumed to be a central pathophysiological cause (Minarova et al., 2023). In order to introduce preventive measures and possible treatments, a better understanding of the exact pathophysiology and mechanisms that cause nephrocalcinosis is required. Previously, we have described the function and role of the kidneys in ion and water regulation in response to salinity (Takvam et al., 2021). Thus, this review aims to **I**) describe the kidneys role in regulation of acid-base equivalents and the two primary ‘urinary buffers’, ammonia and phosphate used by freshwater and marine teleosts, and **II**) integrate known aspects of renal physiology with recent advances in the molecular understanding of membrane transport systems involved in acid-base regulation, ammonia and phosphate excretion via the renal tubules. Where knowledge on these membrane transporters in the teleost kidneys is limited, we will take advantage of mechanisms described for gills and/or the mammalian literature. Our main aim is to integrate recent molecular findings on transport pathways, with classical information on the function of the teleost kidneys in acid-base regulation, ammonia, and phosphate excretion. Finally, how this synthesis applies to pathophysiology and increased prevalence of nephrocalcinosis in modern land-based aquaculture is outlined.

## Factors influencing acid-base balance and excretion of ammonia in fishes

Common factors that challenge acid-base balance in fish include: i) exhaustive exercise (Mommsen and Hochachka, 1988; Wood, 1988; Wood, 1991a) and environmental hypoxia (Kobayashi and Wood, 1980), both of which generate acidic equivalents by adenylate breakdown and lactate production, resulting in metabolic acidosis, sometimes exacerbated by CO_2_ buildup (respiratory acidosis) or ameliorated by CO_2_ washout (respiratory alkalosis) due to hyperventilation; ii) high environmental PCO_2_, often associated with algal respiration, which causes respiratory acidosis, and is a particular issue in closed system aquaculture (Ellis et al., 2017); iii) environmental hyperoxia, often associated with algal photosynthesis, as well as oxygen injection in aquaculture (McArley et al., 2021), which causes hypoventilation and associated CO_2_ retention resulting in respiratory acidosis (Wood and Jackson, 1980; Wood, 1991b); iv) the post-feeding “alkaline tide” (metabolic alkalosis) caused by HCl secretion in the stomach and matching NaHCO_3_ addition to the bloodstream during digestion (Bucking and Wood, 2008; Cooper and Wilson, 2008); v) The post-feeding “acidic tide” in agastric fish caused by NaHCO_3_ secretion in the intestine and matching HCl addition to the bloodstream (Wood et al., 2010); vi) low environmental pH associated with acidic precipitation which induces metabolic acidosis (Wood, 1989); and vii) the decrease in the “strong ion difference” that results in a decrease of plasma HCO_3_^−^ concentration (metabolic acidosis) when freshwater fish move into seawater (Maxime et al., 1991), and increase of plasma HCO_3_^−^ (metabolic alkalosis) that occurs when the transfer is in the opposite direction (Maxime et al., 1990). Some of the factors that challenge acid-base regulation in fish also cause internal ammonia generation and increased ammonia excretion, including i) exhaustive exercise (Wood, 1988); ii) feeding (Karlsson et al., 2006; Bucking and Wood, 2008; Zimmer et al., 2010); iii) freshwater-to-seawater transfer (Wood and Nawata, 2011); iv) exposure to low environmental pH (McDonald and Wood, 1981), whereas v) exposure to high environmental pH inhibits ammonia excretion and causes internal ammonia accumulation (Wilkie and Wood, 1996), and vi) exposure to high environmental ammonia (HEA), another common complication in aquaculture (Beamish and Tandler, 1990; Handy et al., 1993), usually has a biphasic effect, with initial ammonia uptake and then later augmented ammonia excretion by the fish (Nawata et al., 2007; Nawata et al., 2010; Zimmer et al., 2010; Wood and Nawata, 2011).

## Excretion of acid-base equivalents by the kidneys

Relative to the gills, the approximate contribution of the kidneys to the net excretion of acidic equivalents is generally small, based on the studies conducted in teleost fishes so far (Table 1). For example, in freshwater trout, renal excretion accounted for 7% of the compensation during hyperoxia (Wheatly et al., 1984), 16% during hypercapnia (Perry et al., 1987), 5% during the post-feeding alkaline tide (Bucking et al., 2010), 8% during recovery from exhaustive exercise (Wood, 1988), and 12% during recovery from hypoxia-induced lactacidosis (Kobayashi and Wood, 1980; Table 1). In the channel catfish, *Ictalurus punctatus,* the contribution of the kidneys to compensation of some of these same disturbances was greater, but still accounted for less than 33% overall (Cameron, 1980; Cameron and Kormanik, 1982). However, an important exception is the metabolic acidosis of low environmental pH (4.0–4.5) exposure in rainbow trout (*Oncorhynchus mykiss*), where renal excretion of acidic equivalents accounted for 100% of the compensatory response that occurred, as the gills continued to take up acidic equivalents throughout the exposure period (McDonald and Wood, 1981; Wood et al., 1999). Indeed, this treatment induced the highest urinary excretion rate of acidic equivalents ever recorded in a freshwater fish.

**TABLE 1:** Approximate percent (%) contribution to net excretion of acidic equivalents by the gills and kidneys in teleost fishes.

Approximate percent (%) contribution to net excretion of acidic equivalents and ammonia		
Physiological conditions	Kidney (%)	Gills (%)
Hyperoxia	7	93
Hypercapnia	16	84
Post-feeding alkaline tide	5	95
Exhaustive excercise	8	92
Recovery from hypoxia induced lactic acidosis	12	88
Metabolic acidosis caused by environmental acid exposure	100	0
Whole body ammonia excretion	10	90

## Regulation of acid-base equivalents

Under normal circumstances in fasting, resting fish, the net excretion of acid-base equivalents through both the renal and branchial routes is negligible, as the animals are in acid-base balance at this time. However, the kidneys make an incredibly important, often overlooked, contribution to this steady state, by actively reabsorbing the vast majority of the HCO_3_^−^ (i.e., basic equivalents) that has been filtered from the blood plasma into the primary urine in the glomeruli. This is undoubtedly important in both freshwater and seawater teleosts, but quantitatively more so in the former where the glomerular filtration rates (GFRs) are higher. To illustrate this with one example, in the resting freshwater rainbow trout, the filtered load of HCO_3_^−^ was about 48 μmol kg^-1^ h^-1^ (the product of a GFR of about 5.3 mL kg^-1^ h^-1^ and a plasma HCO_3_^−^ concentration of 9 μmol mL^-1^) essentially all of which was reabsorbed in the tubules so that none was excreted (Curtis and Wood, 1992). This represented 13% of the entire plasma HCO_3_^−^ pool of 360 μmol kg^-1^. Without this reabsorption, the entire pool of basic equivalents would have been lost in less than 8 h! Furthermore, when the trout were loaded with excess HCO_3_^−^ by infusion with an isosmotic solution of NaHCO_3_, the HCO_3_^−^ reabsorption rate increased 2.5-fold. A similar increase in the rate of active HCO_3_^−^ reabsorption occurs during the compensation of respiratory acidosis caused be environmental hyperoxia (Wheatly et al., 1984) and environmental hypercapnia (Perry et al., 1987). This ensures that the basic equivalents accumulated via uptake at the gills are not lost through the kidneys. Indeed, in these circumstances, not only do the tubules reabsorb all the HCO_3_^−^ filtered at the glomeruli, they also secrete additional acidic equivalents that are excreted on a net basis. When the respiratory acidosis stimulus is relieved, the HCO_3_^−^ reabsorption and acid secretion mechanisms are rapidly suppressed, while urine flow rate (UFR) continues unchanged, so that large amounts of basic equivalents (HCO_3_^−^), now excess to requirements, can be rapidly excreted via the urine. Interestingly, in the plainfin midshipman (*Porichthys notatus*), an aglomerular marine fish, restoration of normal plasma HCO_3_^−^ levels after suspension of environmental hypercapnia was very slow, presumably reflecting the lack of a direct glomerular filtration route for renal excretion of basic equivalents (Perry et al., 2010).

**FIGURE 1:**
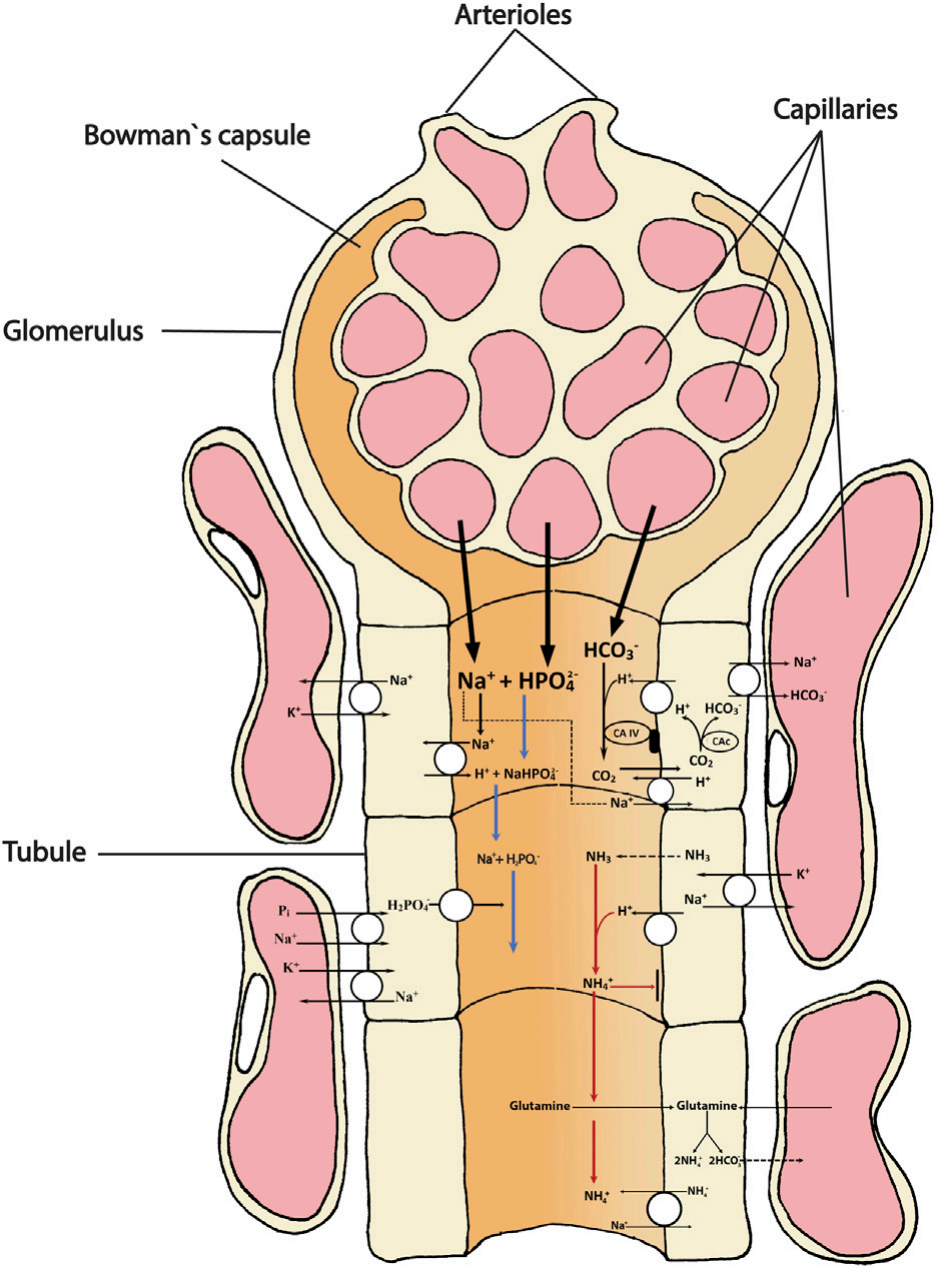
Schematic overview showing the main transport directions of acidic (H^+^, NH_4_^+^) and basic (HCO_3_^-^) equivalents and the uncharged respiratory gases CO_2_ (acidic) and NH_3_ (alkaline) involved in acid-base homeostasis in the teleost kidney. The primary urine filtrate from the glomerulus (and bowman`s capsule) contains bicarbonate (HCO_3_^-^), sodium (Na^+^) and dibasic phosphate (HPO_4_^2-^) that enters the tubule for either excretion or reabsorption as they move along the proximal and distal tubule and collecting duct. Extracellular carbonic anhydrase (CA-IV) on the tubule apical membrane facing the lumen catalyzes the dehydration reaction of a filtered HCO_3_^-^ (appearing in the primary urine) with a H^+^ secreted by the tubule cell. The H^+^ originates from the action of intracellular cytosolic carbonic anhydrase (CAc) that catalyzes the hydration of CO_2_, thereby permitting the HCO_3_^-^ ion to be returned to the blood. The back diffusion of CO_2_ from the urine to the tubule refuels this reaction. The uncharged gases (CO_2_ and NH_3_) move easily across cell membranes, whereas the movements of the charged ions HCO_3_^-^ and NH_4_^+^ must be facilitated by transporters or channels. The two major “urinary buffers” are ammonia and phosphate. Once most of the HCO_3_^-^ has been reabsorbed, any additional H^+^ ions that are secreted stay in the urine, titrating NH_3_ to NH_4_^+^, and dibasic phosphate (HPO_4_^2-^) to monobasic phosphate (H_2_PO_4_^-^), both of which are excreted. Another mechanism of NH_4_^+^ appearance in the urine is also shown and may be quantitatively more important, based on mammalian studies. This is the oxidative metabolism of glutamine (or other amino acids) which produces equimolar amounts of NH_4_^+^ which is secreted into the urine, and HCO_3_^-^ which is returned to the blood plasma. Note that by both of these schemes, for every acidic equivalent excreted, a basic equivalent (HCO_3_^-^) is added to the blood plasma. The different transport mechanisms occur in several segments of the nephron and the model is not intended to illustrate any specific segment, only the mechanisms.

In freshwater fish, the urinary bladder plays an important role in reabsorbing major ions from the tubular urine, thereby contributing to the ion-poor nature of the finally excreted urine, but does not seem to be involved in the addition or removal of acidic or basic equivalents (Curtis and Wood, 1991; Curtis and Wood, 1992). In marine fish, the UFRs are much lower, and urine is held in the bladder for very long periods during which time additional water is reabsorbed and the salts become even more concentrated (Hickman and Trump, 1969; Beyenbach and Kirschner, 1975; Takvam et al., 2021a). Whereas freshwater trout urinate about twice per hour (Curtis and Wood, 1991), marine plaice (*Pleuronectes platessa*) urinate only once every 3–5 days (Fletcher, 1990)! Urine pH is generally very acidic in marine fish, 1.0–2.0 pH units below that of the blood plasma. This probably serves to minimize the precipitation of Ca^2+^ and Mg^2+^ salts in the tubules and bladder during this prolonged residence period, though it is not completely successful (Hickman and Trump, 1969; Miles, 1971). The urinary response to systemic acid-base disturbances appeared to be negligible in two marine teleosts, the shorthorn sculpin *Myoxocephalus scorpius* (Hodler et al., 1955) and the English sole *Parophrys vetulus* (McDonald et al., 1982). Maren et al. (1992) presented an opposing view, but the methods of their experiments on the long-horned sculpin *Myocephalus octodemispinosus* were less than ideal. The low UFRs and the mandatory production of an acidic urine to prevent precipitates are probably important factors limiting the kidneys performance in acid-base regulation in marine fish. Please see illustration on regulation of acid-base equivalents (Figure 1) for a general overview.

## Molecular mechanisms of acid-base regulation in kidneys

In mammals (Weiner and Verlander, 2017), the reabsorption of HCO_3_^−^ depends on the tubular secretion of H^+^ (linked to Na^+^ reabsorption via a Na^+^/H^+^ exchanger and/or a Na^+^ channel coupled to a v-type H^+^ATPase) and is dependent on the enzyme carbonic anhydrase (CA). Intracellular CA catalyzes the hydration of CO_2_ to form the H^+^ ion that is secreted across the apical membrane and the HCO_3_^−^ ion that is returned to the blood across the basolateral membrane, by a Na^+^-HCO_3_^-^ co-transporter. Extracellular CA in the tubule lumen catalyzes the dehydration reaction of the secreted H^+^ with HCO_3_^−^ appearing in the glomerular filtrate. The net effect is that for every one H^+^ ion secreted, one HCO_3_^−^ ion and one Na^+^ ion are removed from the filtrate, while one HCO_3_^−^ (albeit a different HCO_3_^−^) and one Na^+^ ion are returned to the blood. CO_2_ from the dehydration reaction in the urine diffuses back into the renal tubule cell to refuel the H^+^ secretion process. When secreted H^+^ ions exceed filtered HCO_3_^−^ ions, they are buffered in the urine by inorganic phosphate or ammonia, and are excreted, contributing to net acidic equivalent excretion; as a result, a new basic equivalent (HCO_3_^−^) is synthesized on a net basis, and returned to the blood. The whole process is largely powered by Na^+^, K^+^ ATPase (NKA), though v-type H^+^ATPase may also contribute.

All available evidence indicates that the same model applies in fish (Gilmour and Perry, 2009). At the whole animal level, when freshwater trout increase their renal excretion of acidic equivalents, they utilize two major urinary “buffers”, ammonia and phosphate, as in mammals (Hills, 1973; Pitts, 1974). If these were not present, even small amounts of H^+^ secretion in the tubules would drive urine pH so low that the pumping processes would falter. The buffers modulate this; acidic equivalents appear in the urine by the titration of NH_3_ to NH_4_^+^, and the titration of dibasic phosphate (HPO_4_^2-^) to monobasic phosphate (H_2_PO_4_^−^). At least in rainbow trout, NH_4_^+^ appears to be the major buffer mobilized during metabolic acidosis (McDonald and Wood, 1981; Wood et al., 1999) while phosphate predominates during respiratory acidosis (Wheatly et al., 1984; Perry et al., 1987; Wood et al., 1999). In practical terms, the acidic equivalents carried by phosphate can be measured as “titratable acidity (TA)” by titration of the urine back to the blood pH with strong base, while the NH_4_^+^ component, which escapes this titration, is measured directly, and any HCO_3_^−^ in the urine is subtracted, so that net acidic excretion is equal to ([TA]—[HCO_3_-] + [NH_4_^+^]) x UFR (Hills, 1973).

At a molecular level, intracellular CA (cytosolic CAc) has been localized to NKA-rich cells in both proximal and distal tubule cells, whereas extracellular CA (membrane-bound CA IV) occurred in both the apical and basolateral membranes of NKA-rich cells, but only in the proximal tubules (Georgalis et al., 2006). Furthermore, respiratory acidosis induced by environmental hypercapnia increased mRNA of both isoforms with CAc rising quickly and CA IV rising slowly, whereas the protein expression of only CAc increased. Both general CA blockade (with acetazolamide) and selective CA IV blockade (with F3500) increased urinary Na^+^ and HCO_3_^−^ excretion, especially during hypercapnia (Georgalis et al., 2006). Very recently, the salmonid specific CA IV paralogs have been described in the rainbow trout where CA IVb was located in the kidneys, but not the CA IVa (Nelson et al., 2023). The authors point out that several other tissue-specfic differences occur in rainbow trout and highlight the potential divergent roles of the CA IV paralogs in gas exchange and ion/acid-base balance, at least in salmonids. A Na^+^/H^+^ exchanger (NHE3, SLC9A3) and a v-type H^+^ATPase were co-localized to the apical membranes of NKA-rich cells in the proximal tubule of trout (Ivanis et al., 2008), and an anion exchanger (SLC26A1) was found in these same cells (Katoh et al., 2006). The mRNA and protein expression of the NHE3 increased during hypercapnia (Ivanis et al., 2008). Earlier studies had shown that mRNA and protein levels of v-type H^+^ATPase, and mRNA levels of Na^+^-HCO_3_^-^ co-transporter (NBC1) also increased in the trout kidneys during hypercapnia (Perry and Fryer, 1997; Perry et al., 2003a; b).

**FIGURE 2:**
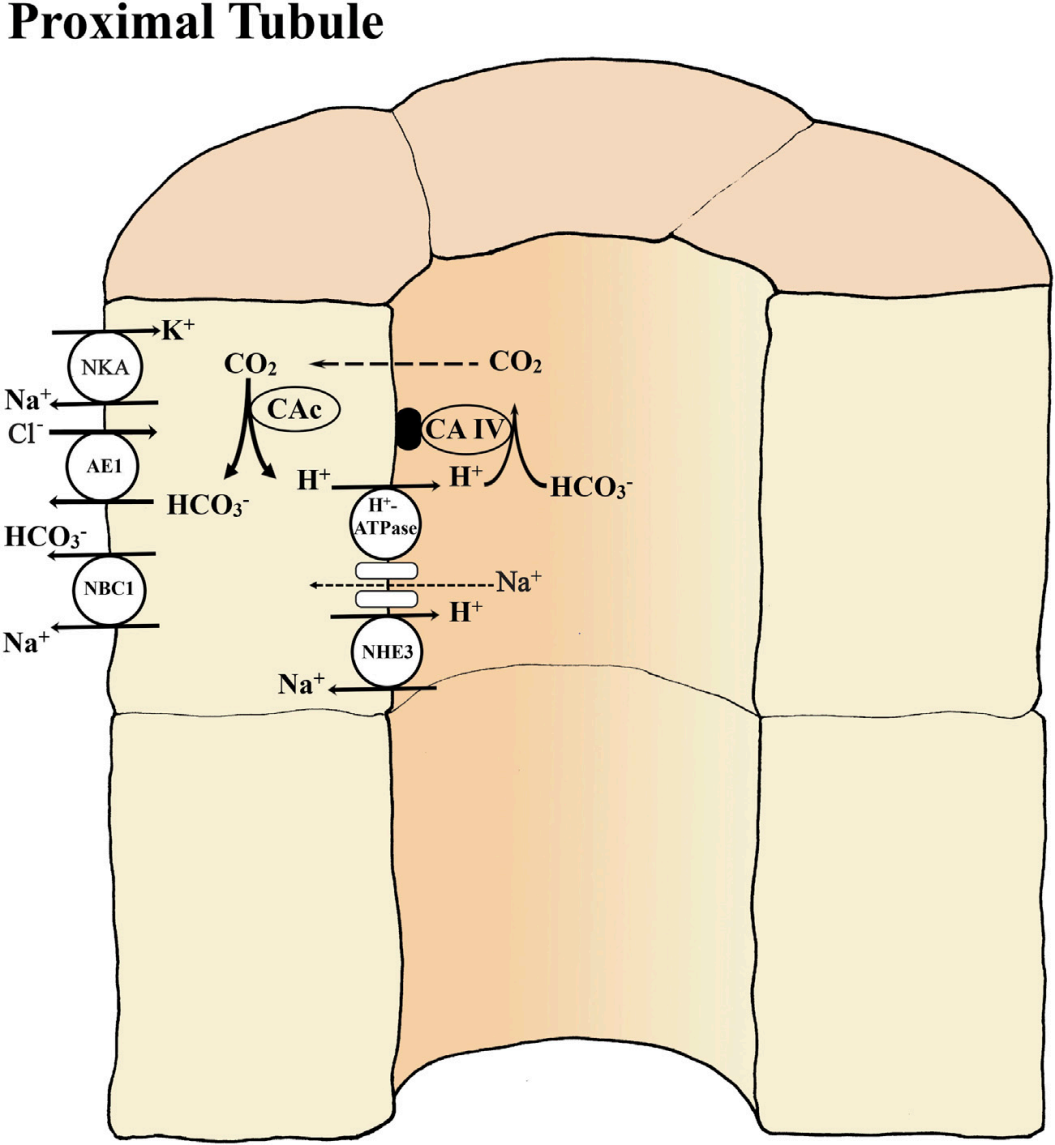
Basic transport mechanisms involved in reabsorption of bicarbonate (HCO_3_^-^) in the proximal tubule of teleost kidney. The basolateral Na^+^- K^+^ -ATPase (NKA) and apical V-type H^+^-ATPase generate favorable transepithelial membrane potentials used by some of the other ion transport pathways. Extracellular carbonic anhydrase (CA-IV) on the tubule membrane dehydrates HCO_3_^-^ in the glomerular filtrate (primary urine) utilizing the H^+^ secreted by an apical Na^+^/H^+^ exchanger (NHE3) and/or by a V-type H^+^-ATPase coupled to a Na^+^ channel. The NHE3 is an abundant but less powerful secondary transport mechanism that cannot secrete H^+^ against low urine pH. The H^+^-ATPase is a less abundant but powerful enzymatic transport mechanism that can secrete against very low urine pH. Inside tubule cells cytosolic (CAc) catalyzes the hydration of CO_2_ forming HCO_3_^-^ to be returned to the blood via a basolateral Na^+^-HCO_3_^-^ co-transporter (NBC1) and/or anion exchanger AE1. If the Na^+^-coupled secretion mechanisms (NHE3, H^+^-ATPase) for H^+^ secretion cannot match the rate of HCO_3_^-^ filtration, then net HCO_3_^-^ excretion occurs, while if the H^+^ secretion rate exceeds the rate of HCO_3_^-^ filtration, then net H^+^ excretion occurs. Note that after all HCO_3_^-^ is reabsorbed continued net H^+^ excretion is dependent on the presence of urinary buffers (see figure 1).

There is only limited support for this “rainbow trout model” in other species. CAc has also been found in proximal tubule cells of marine winter flounder, *Pleuronectes americanus* (Pelis and Renfro, 2004). NHE3 has been found in the cells of the distal nephron of the euryhaline mangrove rivulus, *Kryptolebias marmoratus* (Cooper et al., 2013). However, NHE3 mRNA could not be detected in the kidneys of the European seabass, *Dicentrarchus librax* in either freshwater or seawater (L’Honoré et al., 2020). In the common carp, *Cyprinus carpio*, mRNA expression of v-type H^+^ATPase and NHE3 increased in the kidneys during prolonged metabolic acidosis caused by low environmental pH exposure, but protein expression did not change (Wright et al., 2014). However, these mRNA responses were not seen in goldfish, *Carassius auratus* subjected to similar low pH exposure (Lawrence et al., 2015). Recently profiling of transporters along the length of the nephron in goldfish appears to follow the model previously demonstrated (Fehsenfeld and Wood, 2018). Here the NHE3, v-type H^+^ATPase, CA IIa (cytosolic, similar to CAc of trout), anion exchanger AE1 (similar to SLC26A1 of trout) and NBC1 are all expressed in the proximal tubule and NHE3 is upregulated at both protein and mRNA levels with relocation to the apical membrane, by the metabolic acidosis following feeding in this agastric species. Basolaterally located AE1 is simultaneously downregulated at the protein level in all sections, in accord with less HCO_3_^−^ secretion and greater urine acidification. However, the situation in the goldfish is complicated by findings that v-type H^+^-ATPase is exclusively basolateral in distribution, which fits with observations that goldfish urine is usually highly alkaline under control conditions (Lawrence et al., 2015). Furthermore, expression levels for some of these transporters are comparable or higher in other sections of the nephron (e.g., NHE3 and CAIIa in the distal tubule, connecting tubule, and collecting duct; v-type H^+^-ATPase, AE1, and NBC1 in all sections), with mixed responses to feeding (Fehsenfeld and Wood, 2018). These observations correlate with microelectrode measurements showing that net H^+^ secretion is not localized but occurs along the entire length of the isolated nephron in the goldfish, with elevated rates in all sections following feeding (Fehsenfeld et al., 2019). Interestingly, in this same investigation, blocker studies indicated that v-type H^+^-ATPase was important in the proximal and distal tubule, NHE3 was important in the proximal and connecting tubules, and CA IIa was important in H^+^ secretion in all sections. Studies on a wider range of species are clearly needed. Please see Figure 2 for a detailed overview of current ideas on renal transport of acid-base equivalents.

Given the reduced role of the kidneys acid-base regulation in marine fish discussed earlier, and the general alkalizing effect of transfer to more dilute salinity (Maxime et al., 1990), we might expect that euryhaline fish would increase expression of the renal H^+^ secretion/HCO_3_^−^ reabsorption system when transferred from seawater to more dilute salinity. However, exactly the opposite happened (downregulation of mRNA for v-type H^+^ATPase, CAc, CA IV, NHE3, and NBC1) when 100% seawater-acclimated trout were transferred to 25% seawater (Gilmour et al., 2012). The authors suggested that since intestinal HCO_3_^−^ secretion was reduced, there would be less need for HCO_3_^−^ conservation by the kidneys. Similarly, in European sea bass, mRNA expressions of anion exchangers AE1 and SLC26A6 were downregulated after transfer of seawater-acclimated European sea bass to freshwater (L’Honoré et al., 2020). The opposite response was observed during smoltification and after SW transfer in Atlantic salmon, *Salmo salar* where the salmonid-specific *slc26a6a1* was upregulated in the kidneys (Takvam et al., 2021b). This transporter appears to play an important role in SO_4_^2-^ secretion in the kidneys of euryhaline teleosts (Watanabe and Takei, 2012; Kato and Watanabe, 2016; Takvam et al., 2021a). On the contrary the salmonid-specific *slc26a6a2* was only expressed in FW gills while the *slc26a6a1* was expressed equally in FW and SW intestine (Takvam et al., 2021b). Interestingly, the SLC26A6A has been linked to HCO_3_^−^ transport in the intestine of mefugu, *Takifugu obscurus* (Kurita et al., 2008) and toadfish, *Opsanus beta* (Grosell et al., 2009), while in the gills the same transporter has been linked to both Cl^−^ and HCO_3_^−^ transport in FW acclimated teleosts (Perry and Gilmour, 2006; Deighweiher et al., 2008; Leguen et al., 2015). This emphasize that the SLC26A6 transporter likely performs different tasks and exhibits a broad ion specificity depending on the tissue in question.

## Excretion of ammonia and phosphate by the kidneys

In mammals (Weiner and Verlander, 2017), most of the ammonia entering the urine is produced by the metabolism of amino acids, principally glutamine, in the renal tubule cells; very little enters via glomerular filtration. While all sections of the nephron can produce ammonia, the proximal tubule is the principal site of ammoniagenesis. The substrate glutamine is taken up by specific transporters, both from the urine and from the venous blood. Each NH_3_ diffusing into the urine traps a H^+^ and thereby becomes trapped itself in the lumen (“diffusion trapping”). While this was the original, classical interpretation of the mechanism of urinary buffering by ammonia (Pitts, 1974), modern evidence suggests that it is a minor component in the proximal tubule, and that most ammonia enters as NH_4_^+^ via transporters or channels (Weiner and Verlander, 2017). Nevertheless, the metabolic breakdown of amino acids, most importantly glutamine, produces equimolar amounts of HCO_3_^−^ and NH_4_^+^ on a net basis. For every NH_4_^+^ ion secreted into the urine across the apical membrane, a HCO_3_^−^ ion is added to the blood across the basolateral membrane, and therefore, NH_4_^+^ excreted in the urine represents acidic equivalent excretion, in accord with classical theory (Hills, 1973; Pitts, 1974), regardless of the mechanism by which it entered. Recent research has revealed that further down the tubule, complex recycling and reabsorption of both NH_3_ and NH_4_^+^ occurs in accord with the countercurrent arrangement of mammalian nephrons, resulting in elevated ammonia concentrations in the interstitial fluid. The final net addition of ammonia to the urine (as much as 60%–80% of the total) returns ammonia to the urine from the interstitium, and occurs mainly in the collecting duct, with some contributions by the distal tubule and connecting tubule.

Under normal circumstances in fasting, resting fish, the excretion of ammonia in the urine generally accounts for only a few percent of whole body ammonia excretion, but often a larger fraction of less abundant N-wastes such as urea, creatine, creatinine, uric acid and trimethylamine oxide (Wood, 1993; Wood, 1995; Wood, 2001). Some exceptions exist. In cutthroat (*Oncorhynchus clarki henshawi*) adapted to the high pH (9.4) of Lake Lahontan, the renal contribution to whole body ammonia excretion (10%) was high relative to many other studies on salmonids in freshwater (Wright et al., 2014). In the obligate air-breathing fish, the Amazonian pirarucu, *Arapaima gigas*, which has a relatively low gill area, the kidneys accounted for about 20% of total ammonia excretion (Wood et al., 2020). The few urinary ammonia excretion measurements available for fish in seawater indicate a very low % contribution to whole body efflux (McDonald et al., 1982; Wood et al., 2005). The very low UFRs of marine fish undoubtedly constrain the capacity of the kidneys for ammonia excretion. We are aware of no comparable information on the percentage of phosphate excretion by the kidneys in either freshwater or seawater fish. However, under normal circumstances, the absolute concentration of phosphate in the urine is generally comparable that of ammonia on a molar basis (Curtis and Wood, 1991; Curtis and Wood, 1992; Lawrence et al., 2015). In goldfish injected with exogenous phosphate, Kaune and Hentschel (1987) reported that 65% of the phosphate load was excreted by the kidneys, largely by secretion into the urine. As noted earlier, the urinary excretion rates of both phosphate and ammonia increase markedly during the compensation of respiratory and metabolic acidosis, in accord with the roles of these molecules as key urinary buffers in fish (McDonald and Wood, 1981; Wheatly et al., 1984; Perry et al., 1987; Wood et al., 1999; Lawrence et al., 2015), just as in mammals (Hills, 1973; Pitts, 1974). The phosphate is likely mobilized from bone and scales as deficiencies appear to give bone deformaties (Lall and Kaushik, 2021), whereas ammonia originates from amino acid metabolism (Ip and Chew, 2010). Please see illustration of ammonia and phosphate excretion (Figure 1) for a general overview.

### Molecular mechanisms of ammonia regulation in kidneys

In ureotelic mammals, plasma levels of ammonia are extremely low, so ammonia enters the urine almost entirely by secretion. However, the molecular mechanisms of ammonia transport remain controversial and incompletely understood (Weiner and Verlander, 2017). In the proximal tubule, apical NHE3 seems to play a predominant role, with NH_4_^+^ substituting on the H^+^ site, such that NH_4_^+^ secretion is coupled to Na^+^ reabsorption. NH_4_^+^ movement through apical K^+^ channels, as well as the classic “diffusion trapping” mechanism, with NH_3_ diffusion coupled to apical H^+^ secretion by NHE3 and/or v-type H^+^ATPase, may also contribute, again facilitating coupling to Na^+^ reabsorption. The sodium, potassium, two chloride cotransporter type 2 (NKCC2) seems to be the principal reabsorptive transporter contributing to ammonia recycling in the loop of Henle, with NH_4_^+^ substituting on the K^+^ site. The final net addition of ammonia in the collecting duct seems to be largely mediated by two Rhesus (Rh) glycoproteins, which are channels that facilitate the diffusion of ammonia. Rhbg, which is exclusively basolateral, serves for basolateral uptake from the interstitium into the tubular cells, while Rhcg, which is both apical and basolateral, serves for apical secretion while supplementing basolateral uptake. Controversy continues as to whether the Rh proteins facilitate NH_3_ movement (perhaps true for Rhcg), NH_4_^+^ movement, or both (perhaps true for Rhbg). NH_4_^+^ substitution on the K^+^ site of basolateral NKA also contributes to basolateral uptake in the collecting duct. HCN2, a hyperpolarization-activated cyclic nucleotide gated channel (subtype 2) has also been implicated in basolateral NH_4_^+^ uptake. On the apical membrane, the “diffusion trapping mechanism” applies, with v-type H^+^ATPase as well as H^+^, K^+^ ATPase providing the apical H^+^ secretion (and perhaps also direct NH_4_^+^ transport by the H^+^ site), while Rhcg facilitates the apical diffusion of NH_3._

In ammoniotelic fish, plasma ammonia concentrations are higher than those in ureotelic mammals, but still very low (micromolar) relative to urinary levels (millimolar). Therefore, as in mammals the great majority of ammonia enters the urine by tubular secretion rather than glomerular filtration, probably produced in the tubule cells by deamination and deamidation of glutamine and other amino acids. In a range of freshwater species, urine/plasma ratios of ammonia are high, and increase further during compensation of acidosis, indicating increased secretion (Cameron and Wood, 1978; McDonald and Wood, 1981; Wood et al., 1999; Wright et al., 2014; Lawrence et al., 2015; Thin et al., 2019). Renal activities of potential ammoniagenic enzymes (phosphate-dependent glutaminase, glutamate dehydrogenase, ɑ-ketoglutarate dehydrogenase, alanine aminotransferase, aspartase aminotransferase, phosphoenolpyruvate carboxykinase) are substantial, and some increase during experimental acidoses (Walton and Cowey, 1977; 1982; King and Goldstein, 1983; Wood et al., 1999; Lawrence et al., 2015), as well as mRNA for glutamate dehydrogenase (Hirata et al., 2003; Fehsenfeld and Wood, 2018). At least in the goldfish, glutamate dehydrogenase mRNA is expressed in all sections of the nephron, increasing during acidosis, and with highest levels in the proximal tubule, again as in mammals (Fehsenfeld and Wood, 2018). Given the lack of a countercurrent morphology, it seems likely that the ammonia transport mechanisms in fish will not necessarily parallel those in mammals. Nevertheless, the same basic elements appear to be present, with many similarities.

**FIGURE 3:**
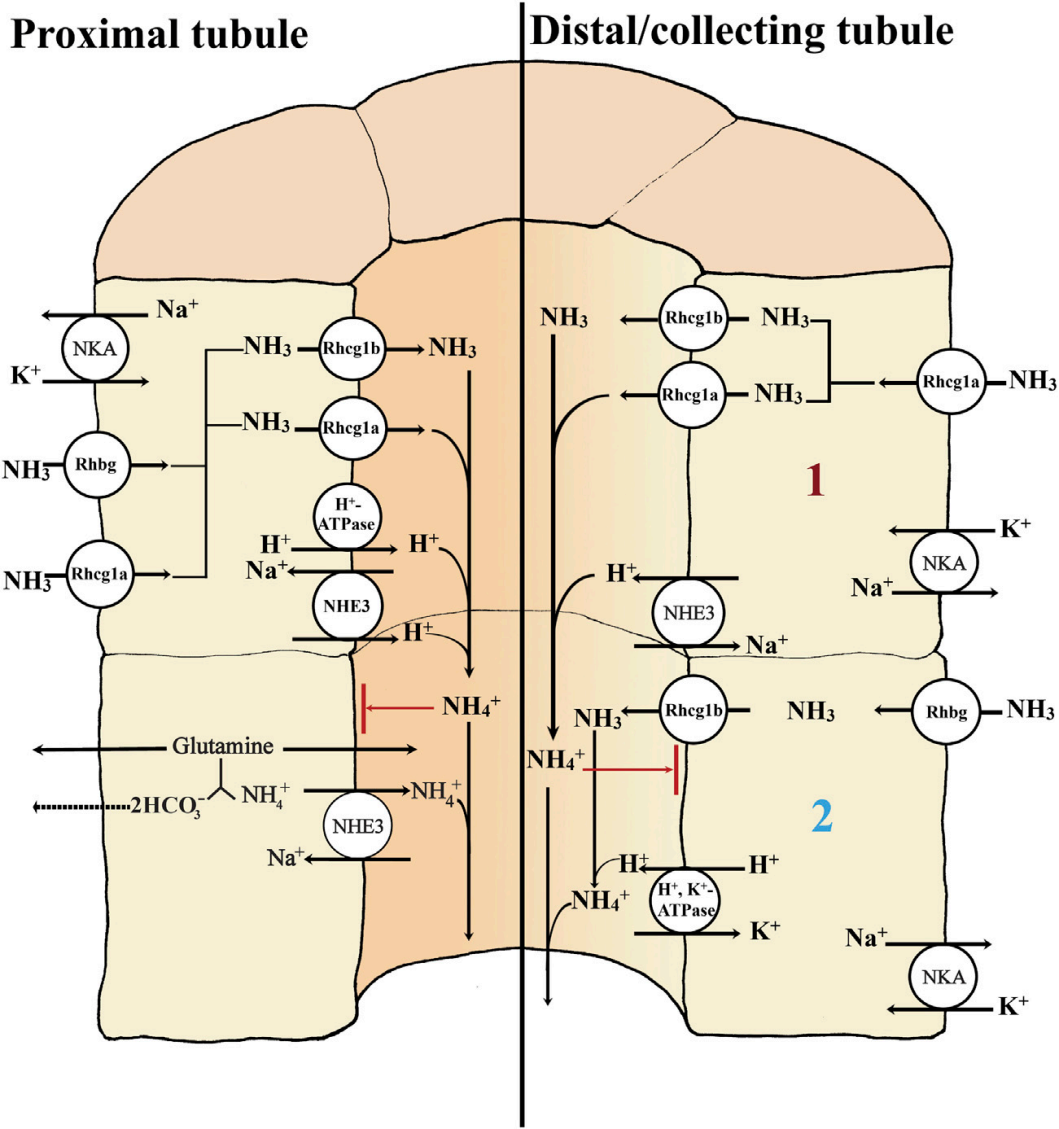
Basic transport mechanisms involved in secretion of ammonia in the proximal and distal/collecting tubule of teleost kidney. The basolateral Na^+^- K^+^ -ATPase (NKA) and apical V-type H^+^-ATPase generate favorable transepithelial membrane potentials used by some of the other ion transport pathways in the proximal and distal/collecting tubule (left side and right side; 1 and 2). The rhesus (Rh) glycoproteins are channels that facilitate the diffusion of ammonia; although NH_3_ is shown as the form moving through them, there is considerable uncertainty as to whether they facilitate the movement of NH_3_, NH_4_^+^, or both. Rhbg and Rhcg1a enable the basolateral uptake of ammonia. Secretion of NH_3_ into the proximal tubule lumen is facilitated by apically located Rhcg1b and/or Rhcg1a channels (left side). Apical V-type H^+^ ATPase (proton pump) and NHE3 (Na^+^/H^+^ exchanger) both may transport H^+^ to the lumen, enabling the “diffusion trapping” mechanism (NH_3_ + H^+^) to form NH_4_^+^. The diffusion trapping mechanism is important to avoid NH_3_ reabsorption back to the tubule cells and maintains the gradient for NH_3_ secretion, ensuring that NH_4_^+^ can ultimately be excreted through the urine. In the proximal tubule, NHE3 may play a dominant role, not only exchanging H^+^ against Na^+^ for diffusion trapping but also directly exchanging NH_4_^+^ (from the oxidation of glutamine and other amino acids) for Na^+^ (left side). The NHE3-mediated diffusion trapping mechanism is also found in the distal/collecting tubule; there, the Rhcg1a channels perform the basolateral uptake of ammonia (right side; 1). However, in the distal/collecting tubule a second mechanism is present (right side; 2). Here the basolateral uptake of ammonia is possible through the Rhbg channel and apical secretion via the apical Rhcg1b. The diffusion trapping mechanism is possible via the H^+^, K^+^ ATPase, providing the apical H^+^ secretion needed to form NH_4_^+^ for later excretion in the urine. Both NH_4_^+^ substitutions on the K^+^ site of the basolateral Na^+^, K^+^ ATPase and a HCN2 (a hyperpolarization-activated cyclic nucleotide gated channel (subtype 2)) have also been hypothesized as a possible NH_4_^+^ basolateral uptake mechanisms in the collecting duct (not shown).

As noted earlier, one parallel is the presence of NHE3 and v-type H^+^ATPase in the proximal tubule of trout (Ivanis et al., 2008) and goldfish (Fehsenfeld and Wood., 2018). Furthermore, in the kidneys of the freshwater Nile tilapia *Oreochromis niloticus*, H^+^, K^+^ ATPase mRNA is expressed, with apical protein localization only in NKA-rich cells of the collecting tubule, another clear parallel to the mammalian model (Barnawi et al., 2020). Rhbg is expressed at the mRNA level in the kidneys of freshwater rainbow trout (Nawata et al., 2007) and the mangrove rivulus, *K. marmoratus* (Cooper et al., 2013); in the latter, there was apical co-localization of the protein with NHE3 in distal tubule cells. However, neither Rhbg nor Rhcg transcripts were detected in the kidneys of the pufferfish (Nakada et al., 2007a). Conversely, in both goldfish (Lawrence et al., 2015) and common carp (Wright et al., 2014), Rhcg (two isoforms) and Rhbg are present. Rhcg1a mRNA and protein were upregulated in response to the metabolic acidosis of low environmental pH exposure in the carp, whereas Rhcg1b mRNA responded in the same way in the goldfish. In the carp, Rhbg mRNA decreased during acidosis, though protein expression did not change (Wright et al., 2014). More detailed profiling in the goldfish revealed that Rhbg protein was basolaterally located, Rhcg1b was apical, whereas Rhcg1a was expressed in both membranes, in both proximal and distal tubules (Fehsenfeld and Wood, 2018). Rhcg1b mRNA and protein were both upregulated in the distal tubule, connecting tubule and collecting duct in response to the metabolic acidosis associated with feeding, whereas Rhcg1a mRNA and protein showed a more modest response in the distal tubule only. Rhbg mRNA was upregulated in the connecting tubule and collecting duct, but downregulated in the distal tubule, accompanied by decreased expression of the protein (Fehsenfeld and Wood, 2018). There was also strong expression of Rhcg1 in the kidneys of the zebrafish at both mRNA and protein levels, with localization to the apical membrane in the collecting duct and distal tubule (Nakada et al., 2007a). Please see Figure 3 for a detailed overview of current ideas on renal transport mechanisms in ammonia regulation.

With respect to other putative ammonia transport mechanisms seen in mammals, various HCN genes, including HCN2b are expressed at the mRNA level in the goldfish kidneys, and several (though not HCN2b) respond to feeding and/or HEA exposure (Fehsenfeld and Wood, 2020). NKCC2 (the principal NH_4_^+^ transporter of the loop of Henle in mammals) is detectable at the mRNA level in the kidneys of seawater acclimated European sea bass, but is downregulated after transfer to freshwater (L'Honoré et al., 2020), so it is unclear whether it is involved in NH_4_^+^ transport. NH_4_^+^ substitution on the K^+^ site of renal NKA probably occurs, as in kidneys of the Amazonia pirarucu, NH_4_^+^ was more effective than K^+^ in activating the activity of this enzyme (Hochachka et al., 1978). Interestingly, in this obligate air-breather, unusually high urinary ammonia excretion was apparently coupled to high urinary HCO_3_^−^ excretion (Wood et al., 2020). Studies of the specific mechanisms of ammonia regulation in the teleost kidneys are limited to a few number of species to date. Hence, both the localization and specific role of Rh proteins are still under debate and more research is required.

### Molecular mechanisms of phosphate regulation in kidneys

A comprehensive review of phosphate handing by teleosts, with a particular focus on the kidneys, has been presented by Verri and Werner (2019). Unlike ammonia, phosphate is originally obtained mainly from the diet and stored in the bones (Vielma and Lall, 1998). Plasma phosphate levels (millimolar) are much higher than ammonia levels (micromolar). Under control conditions, phosphate filtered at the glomeruli is normally strongly reabsorbed by the tubules in freshwater fish (Vielma and Lall, 1998), but during experimental acidosis, this reabsorption slows and may change over to tubular secretion (Wheatly et al., 1984; Perry et al., 1987; Wood et al., 1999; Lawrence et al., 2015). Tubular secretion may normally dominate in marine teleosts. In the marine winter flounder, *Pseudopleuronectes americanus*, secretion is driven by a type-II sodium-phosphate cotransporter (NaPi-II), now termed Slc34a2b, that is sorted to the basolateral membrane of the second part of the proximal tubule, whereas in the collecting tubule and collecting duct, the same transporter is located in the apical membranes and appears to drive reabsorption (Gupta and Renfro, 1989; Elger et al., 1998; Verri and Werner, 2019). Low peritubular pH strongly stimulated tubular phosphate secretion in an *in vitro* culture system (Gupta and Renfro, 1991). Both mRNA and protein for a reabsorptive NaPi-II cotransporter (now slc34a1) were identified in the kidneys of the freshwater rainbow trout, only in the first part of the proximal tubule, with localization to the apical membrane (Sugiura et al., 2003). This transporter is upregulated in response to low dietary phosphate in trout (Coloso et al., 2003; Sugiura and Ferraris, 2004), but we are aware of no information on its response to acid-base disturbance.

## Relevance for aquaculture

**FIGURE 4:**
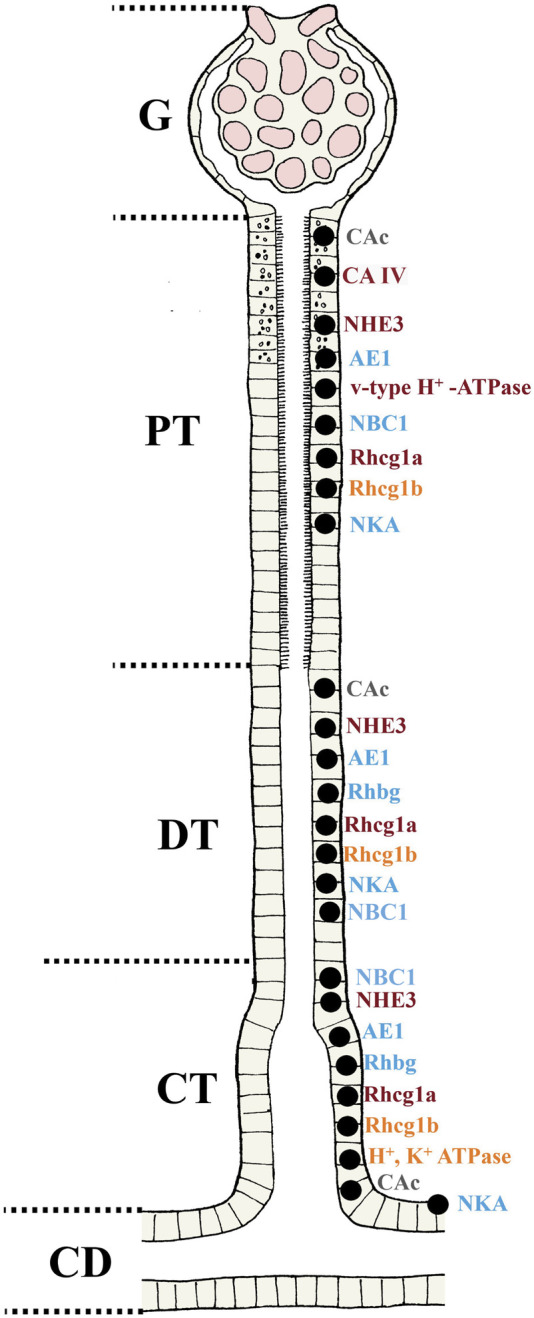
Overview of the localization of the transporters involved in acid-base, ammonia and phosphate regulation in teleost fish. Based on the current understanding and molecular analyses in teleosts, putative locations of various transporters in the nephron are presented for fish (G; Glomerulus, PT; Proximal tubule, DT; Distal tubule, CT; Collecting tubule and CD; Collecting duct). For the regulation pattern in different situations (e.g. changes in acid-base status, water chemistry, fasted versus non-fasted states) the reader is referred to the text. The CAc are found in all sections of the nephron PT, DT and CT. While similar location has been found for the CA IV it is not present in the CT. The NHE3, NBC1 AE1, Rhcg (1a and 1b), Rhbg and NKA are found in all segments. The V-type H^+^ ATPase are found only in the PT, and the H^+^, K^+^ ATPase only in the CT. Apical (orange), basolateral (blue) and multiple locations (dark red) are shown. The CAc (grey) is found in the cytosol of the cell.

Based on the preceding material, we can conclude that the kidneys plays an important (and often overlooked) role in the regulation and removal of acid-base equivalents and ammonia in fish, especially in freshwater. Overall, the mechanistic details of acid-base regulation and ammonia excretion by the renal tubules of teleost fish are clearly complex and remain elusive, especially at the gene and molecular level (see Figure 4; locations of known transporters in the nephron). However, there are many aspects of the regulation that are understood at the macro level, where the integration of emerging genetic and molecular methods could further illuminate the mechanisms involved (see Future Research Direction). Traditionally, some of the most devastating diseases attacking fish in aquaculture have been kidney diseases—for example, proliferative kidney disease (PKD; Hedrick et al., 1993), bacterial kidney disease (BKD; Fryer and Sanders, 1981), nephrocalcinosis (Fivelstad et al., 2018), and hemorrhagic smolt syndrome (HSS; Byrne et al., 1998; Krasnov et al., 2019), all of which have severe fish health and welfare consequences. Knowledge of the kidney’s roles in acid-base, ammonia and phosphate regulation will be vital to better understand how the kidneys respond to changes in water quality and feed composition but also in kidney disease. This will be important especially in land-based intensive farming. Although these aspects definitely applies to other cultivated species in land-based aquaculture the main focus will be on Atlantic salmon and Rainbow trout as perturbations in the kidney is one of the leading welfare issues during land-based rearing of these species.

### Water chemistry in land-based aquaculture systems and implications for renal physiology

Since the mid-2000, investments in larger and technologically more sophisticated recirculating aquaculture systems (RAS) have increased significantly, with the emergence of new production related challenges and risks (Daalsgard et al., 2013; Fossmark et al., 2021; Davidson et al., 2023). Recycling water permits reduced consumption of intake water per unit biomass produced, constant high temperature, use of continuous light and increased salinity which has resulted in increased average smolt size, from 50 to 100 g in 2008 to approximately 300–500 g in 2022, with several companies aiming to produce smolts up to 600–800 g and future prospects of a 1.000 g fish (Ytresøyl et al., 2023). Despite RAS facilities providing greater control of rearing conditions, the industry has experienced increased incidents of disease-related production disorders such as mineral precipitation in the kidneys (Fivelstad et al., 2018; Klykken et al., 2022a; Klykken et al., 2022b) and hemorrhagic smolt syndrome (Nylund et al., 2003; Krasnov et al., 2019), which have negative implications for growth, health and general performance during the land-based phase (Sommerset et al., 2022). These production disorders are likely a consequence of the rapid changes in technology from flow-through systems (FTS) to RAS, and associated with this, altered rearing protocols for more intensive production (changes in photoperiod, respiratory gas levels, temperature, salinity, stocking density, feed composition and use of buffers and alkalizing reagents). These may ultimately change the overall chemistry of the culture water to sub-optimal conditions which in turn may result in physiological perturbations. However, nephrocalcinosis appear to an issue also in FTS systems (Klykken et al., 2022a). Oxygenation of rearing water without removal of accumulated CO_2_ combined with high fish density and potentially low water renewal rate may result in increased blood PCO_2_ and respiratory acidosis associated with both hypercapnia and hyperoxia (Skov, 2019; McArley et al., 2021). Complex interactions occur between pH, temperature and salinity and the formation of carbonate compounds, often referred to as the carbonate system. This is normally affected by the partial pressure of dissolved carbon dioxide (PCO_2_), and the concentrations of bicarbonate ion (HCO_3_^−^), carbonate (CO_3_^2-^) ion, hydrogen ion (H^+^) (usually expressed indirectly as the negative logarithm, pH), dissolved inorganic carbon (DIC) and the total alkalinity (TA).

Moving juvenile fish from FTS to RAS gives a larger degree of control, yet more demanding technology and complex interactions in pH, temperature, alkalinity, oxygen saturation and suspended solids, which in turn have direct consequences on dissolved total CO_2_ (DIC), nitrogen, ammonia and nitrate (Daalsgard et al., 2013; Becke et al., 2020; Fossmark et al., 2021; Davidson et al., 2023). In addition, the use of salinity (in feed or water) has implications on kidney function (Hickman and Trump, 1969; Kodzhahinchev et al., 2017) and the cardiovascular system (Olson and Hoagland, 2008; Brijs et al., 2017; Sundell et al., 2018). In humans these systems are intrinsically connected, and the same is probably true in fish. For example, chronic kidney disease (CKD) impacts the cardiovascular system and increases the risk of heart failure (Said and Hernandez, 2014; Mullens et al., 2020). We do not yet know whether chronic long-term nephrocalcinosis has similar impacts on the cardiovascular system in fish.

### Nephrocalcinosis and renal pathophysiology

Nephrocalcinosis, a disease of aquaculture that has been known for almost 50 years (Landhoilt, 1975), is a renal pathology where a mechanistic understanding of the kidney`s function in acid-base regulation will be critical. Nephrocalcinosis is a widespread disorder found in salmonids (Gillespie and Evans, 1979; Harrison and Richards, 1979; Smart et al., 1979; Fivelstad et al., 1999; 2003; 2018; Klykken et al., 2022a; Minarova et al., 2023), Nile tilapia (*Oreochromis niloticus;* Chen et al., 2001; 2003), sea bass (*Dicentrarchus labrax;* Arciuli et al., 2015), Atlantic cod (*Gadus morhua*; Damsgard et al., 2011), spotted wolffish (*Anarhichas minor*; Foss et al., 2003; Béland et al., 2020), clownfish (*Amphiprion ocellaris*; Blazer and Wolke, 1983), cobia (*Rachycentron canadum*; Klosterhoff et al., 2015), southern flounder (*Paralichthys lethostigma;* Appelgate et al., 2016), Atlantic wolffish (*Anarhichas lupus*; Béland et al., 2020), longsnout seahorse (*Hippocampus reidi* Ginsburg; Lewisch, et al., 2013), lumpfish (*Cyclopterus lumpus*) and ballan wrasse (*Labrus bergylta*) (Erkinharju et al., 2021), and is listed as one of the most prevalent perturbations in Norwegian salmonid culture, particularly with respect to land-based productions of large smolt and post-smolts (Sommerset et al., 2022). The physiological perturbations underlying precipitation of minerals in the kidneys are probably related to elevated concentrations in the diet or in the water of the constituents involved in forming the precipitate, principally calcium, magnesium, and phosphate (Smart et al., 1979), and/or because changes in the water chemistry cause changes in internal physiology which promote the formation of these “kidney stones”. Bacterial and viral pathogens are probably not the primary cause (Landholt, 1975), but there is a need to characterize the microbiome associated with the precipitates, as well as their precise mineral composition in fish raised in culture waters of different composition and fed diets of different composition. According to Klykken et al. (2022a), there are at least 5 different chemical compositions for kidney stones in Atlantic salmon, involving precipitates of calcium, magnesium, phosphate, and carbonate. The most prevalent was amorphous carbonate apatite (Ca_10_(PO_4_)_6_CO_3_).

In mammals the carbonate apatite (Ca_10_(PO_4_)_6_CO_3_) forms and precipitates at a pH ≥ 6.8 indicating that there is a specific pH threshold in the pre-urine/urine that needs to be sustained in order for stones to form or not form (Olszynski et al., 2015). Similarly, struvite (MgNH_4_PO_4_ 6H_2_O) is commonly found in both mammals and salmonids alike (Olzinsky et al., 2015; Klykken et al., 2022a), and in mammals this appears to form and precipitate at pH ≥ 7.2 (Olzinsky et al., 2015). As noted earlier, urine pHs are generally higher in FW fish (similar to blood plasma pH, 7.0—8.0) while the urine pH is more acidic in marine fish, 1.0–2.0 pH units below that of the blood plasma (Hickman and Trump, 1969). This may be one reason why nephrocalcinosis generally has been observed to be reduced when salmonids have been transported to SW (Klykken et al., 2022a). However, little is known about the specific pH threshold for stone formation in the urine of salmonids. More studies are required to better understand changes in urine pH, but the interpretation of such results should be done with caution as pH changes in the final urine may not reflect pH changes locally in the different segments of the nephron where stones likely starts to form. *In vitro* experiments recreating a similar environment (artificial urine), similar to those performed by Olzinsky et al. (2015), are needed in fish. Environmental changes that increase the urinary pH of salmonids may be a significant risk factor, as higher pH in mammals definitely increased precipitation events of both carbonate apatite and struvite.

**FIGURE 5:**
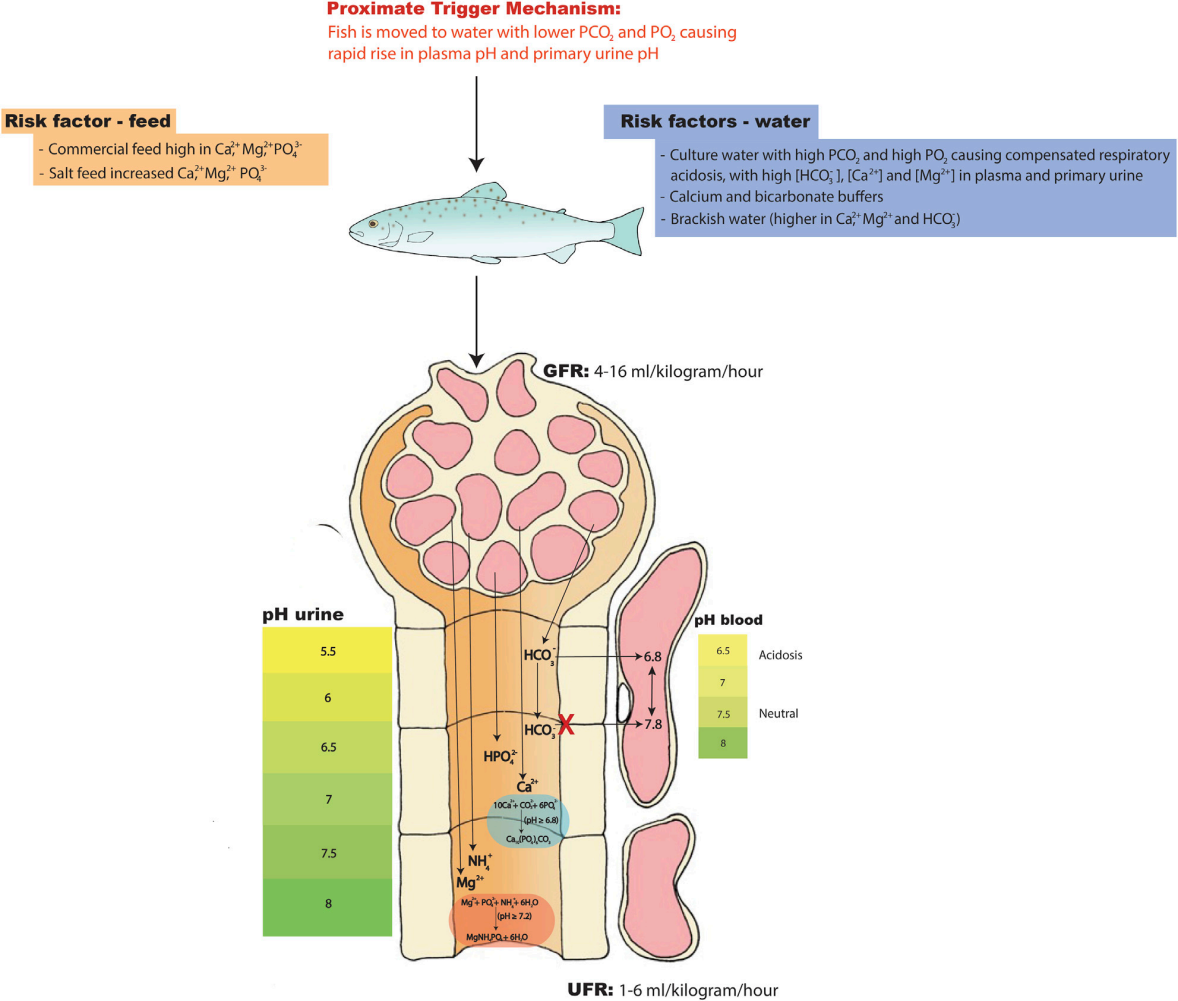
Hypothesis on the physiological and molecular mechanisms leading to the formation and aggregation of kidney stones in nephrocalcinosis. During elevated PCO_2_ and PO_2_ levels fish will experience chronic respiratory acidosis where blood pH is reduced and where the pH has been compensated by HCO_3_^-^ retention for which the kidney tubules have a key compensatory role. If water PCO_2_ and PO_2_ drop in the tank then PCO_2_ in the blood plasma and primary urine will quickly fall while HCO_3_^-^ in the plasma and primary urine will stay high for some time. The pH will quickly rise in the blood plasma and primary urine during this period, creating optimal conditions for nucleation, precipitation, and aggregation events to occur in urine that still has elevated Ca^2+^, Mg^2+^, NH_4_^+^, HPO_4_^2-^ and HCO_3_^-^ concentrations. During this event the kidney stones will start forming at pH above 6.8 (carbonate apatite; Ca_10_(PO_4_)_6_CO_3_) and 7.2 (struvite; MgNH_4_PO_4_ ·6H_2_O). This may be the proximate trigger mechanism. Increased salt load in the water (12-15 ppt), salted feed and/or calcium and sodium bicarbonate buffers may further increase the Ca^2+^, Mg^2+^ and HCO_3_^-^ having a cumulative effect. For more details regarding the transport pathways the reader is referred to Figure 1, Figure 2, Figure 3.

The association between nephrocalcinosis and elevated water PCO_2_ (hypercapnia) was originally documented by Smart et al. (1979) and has now been confirmed by numerous studies (e.g.,; Fivelstad et al., 1999; 2003; Foss et al., 2003; Hosfeld et al., 2008; Petochi et al., 2011). A recent review (Skov, 2019) outlined effects of environmental hypercapnia in fish culture. Elevated PCO_2_ in the production water will initially affect oxygen transport and acid-base regulation, which in turn may result in increased prevalence of nephrocalcinosis. When hypercapnia is combined with hyperoxia, the incidence of nephrocalcinosis increases, presumably due to greater PCO_2_ build-up in the fish (Wood and Jackson, 1980; Hobe et al., 1984; Wood, 1991b; Skov, 2019), which causes respiratory acidosis. In turn, the fish compensates by increasing the concentrations of inorganic phosphate, total ammonia, and acidic equivalents in the finally excreted urine, thereby retaining and raising blood plasma HCO_3_^−^ concentrations to compensate the respiratory acidosis as outlined earlier (Wood and Jackson, 1980; Wheatly et al., 1984; Perry et al., 1987; Wood, 1991b; Wood et al., 1999). Urine pH falls only slightly due to the buffering action of ammonia and phosphate, and urinary calcium and magnesium concentrations increase only slightly (Wheatly et al., 1984; Perry et al., 1987). A recent diagnostic case study reported 5-fold greater PCO_2_ and 10-fold greater HCO_3_^−^ concentration in the blood plasma of rainbow trout that were afflicted with a high incidence of nephrocalcinosis in an aquaculture facility with water hypercapnia and hyperoxia (Minarova et al., 2023). Hence, while HCO_3_^−^ concentrations may not be high in the finally excreted urine, they may be excessively high in the primary urine formed by glomerular filtration, as well as in the surrounding peritubular blood plasma. This, combined with greatly elevated PCO_2_, ammonia and phosphate, and slightly elevated calcium and magnesium concentrations in the urine may be important for mineral precipitates to form and grow. A model, incorporating this idea, is offered in the next section and Figure 5.

Although environmental hypercapnia causes acid-base perturbations and is a well-established risk factor for development of nephrocalcinosis in farmed salmon (Skov, 2019), it is clearly not the only culprit. Atlantic salmon post-smolts exposed to high total CO_2_ levels (5–40 mg L^-1^; see Skov et al. , 2019, for conversion to PCO_2_ units) and brackish water (12 ppt) for 12 weeks did not exhibit any signs of nephrocalcinosis until after 6 weeks in full strength seawater (<5 mg L^-1^ CO_2_; 34 ppt) (Mota et al., 2019; 2020). Similarly, Good and co-authors (2018) found no evidence of nephrocalcinosis in Atlantic salmon exposed to 20 mg L^-1^ CO_2_ for 384 days. Salmon in the latter study experienced higher water alkalinity (233–241 mg L^-1^) and freshwater conditions, while seawater alkalinity ranged between 116 and 165 mg L^-1^ in the study by Mota and co-authors (2019). Both studies used NaHCO_3_ buffer to maintain alkalinity. Interestingly, Nile tilapia reared in water buffered with NaHCO_3_ exhibited higher plasma HCO_3_^−^ concentrations and only modest prevalence of nephrocalcinosis whilst tilapia reared in FW buffered by CaCO_3_ displayed the opposite response, low plasma HCO_3_^−^ concentrations and high prevalence of nephrocalcinosis (Chen et al., 2001). Hence, both salinities and the specific composition of the ions in the water may affect the prevalence of nephrocalcinosis differently. Divalent cations (Ca^2+^, Mg^2+^) and the divalent anion (HPO_4_^2-^) in the water and/or feed are probably important risk factors. Elevated Ca^2+^ and Mg^2+^ are often a result of adding seawater to increase rearing salinity (Table 2). Additionally, the use of calcium-containing buffers and/or increased salt load via the feed may have osmoregulatory impacts, including kidney dysfunction. How much of an impact depends on type of ions and concentrations and the manner in which the fish are exposed (i.e., via the feed or via the water).

**TABLE 2:** General overview of water quality parameteres (ranges) in modern freshwater (FW) and brackish water (BW) recirculating aquaculture systems (RAS) in Norway.

Parameters	Units	RAS-FW	RAS-BW
pH		6.8 - 7.2	7.6 - 7.7
PCO2	**mg/L**	12.6 - 20.6	1.0 - 14.0
Oxygen saturation	**percent (%)**	77 - 111	
Conductivity	**mS/m**	328 - 12	1750 - 1770
Alkalinity (CaCO3)	**mg/L**	25.2 - 33.8	94.5 - 110
Salinity (calculated based on Chloride)	**ppt**	0.01	11.1 - 11.6
Ammonium (NH4-N)	**mg/L**	0.09 - 0.14	0.1 - 13.4
Ammonia (NH3-N), calculated	**mg/L**	0.05 - 0.20	0.001 - 0.134
Total Organic Carbon (TOC)	**mg/L**	2.0 - 4.8	13.8 - 15.4
Chloride (Cl-)	**mg/L**	6.05 - 7.3	6000 - 6500
Calcium (Ca2+)	**mg/L**	0.22 - 6.6	140 - 150
Magnesium (Mg2+)	**mg/L**	0.6 - 0.71	420 - 430
Sodium (Na+)	**mg/L**	18 - 42	3700 - 3900

In mammals, low GFR has been identified as one of several causes underlying the formation of kidney stones (Gillen et al., 2005; Worcester et al., 2006) but also high dietary salt and protein intake (Ferraro et al., 2020). Interestingly, GFR in freshwater acclimated fish is high, producing copious amounts of dilute urine (i.e., high UFR), while GFR in seawater acclimated fish is lower, resulting in low UFR of a urine rich in divalent ions (Hickman, 1968a; 1968b; Takvam et al., 2021a). Based on the mammalian model (Gillen et al., 2005; Worcester et al., 2006; Ferraro et al., 2020), the expectations would be that more salinity (through feed and water) would reduce GFR and be a risk factor for developing nephrocalcinosis. Commercial diets often contain high salt levels (Na^+^, Cl^−^, Ca^2+^, Mg^2+^ and inorganic phosphate). Furthermore, commercial feed given to fish in the freshwater stage usually contains ingredients from marine sources which increases the salt load (Shaw et al., 2011; Phillip et al., 2022). From a biological standpoint, this is unnatural for fish in freshwater. In freshwater, fish usually drink very little. Increased salinity of the water will increase drinking rate, though the threshold for this effect is unclear (Damsgaard et al., 2020), and increased salt content of the food will have the same effect (Pyle et al., 2003). Interestingly in mammals, higher salt intake appears to be a big risk factor while drinking adequate levels of water appears to somewhat offset the risks of developing kidney stones (Ferraro et al., 2020). How many of these mechanisms in the mammalian model that can be translated to the fish model is unclear, but it is noteworthy that nephrocalcinosis in euryhaline salmonids, has been observed in both freshwater and in brackish water. Although brackish water (12–15 ppt) has been pointed out as a potential risk factor (Klykken et al., 2022a), other triggers are in play earlier in the production as farmers report incidences of nephrocalcinosis down to 7 g fish which may point to components in the feed. Future research should address these interactions between salt load (water and feed), urine production rates (GFR and UFR) and link this to water chemistry levels discussed here at various specific life-cycle stages during production. Yet, the proximate mechanism by which nephrocalcinosis develops in teleost fishes remains unknown almost 50 years after its first discovery (Landholt, 1975).

In light of our understanding of both ion and water homeostasis (Takvam et al., 2021a) and acid-base balance (current review), we suggest a new model for this proximate mechanism (Figure 5). Increased risk of nephrocalcinosis may be coming from the increased salt load in feed (commercial diets; high in Mg^2+^, Ca^2+^ and PO_4_^2-^) and the addition of buffers in the water such as calcium hydroxide (Ca(OH)_2_) and sodium bicarbonate (NaHCO_3_) (most commonly used buffers in RAS) in the early freshwater phase. However, we suggest that the key condition for kidney stone formation may start when fish are experiencing elevated PCO_2_ and PO_2_ levels in the water of their holding tanks. These fish will be in the condition of a chronic respiratory acidosis where the pH has been compensated by HCO_3_^−^ retention in the body fluids, which is achieved by excretion of acidic equivalents in the urine (see Figures 1–3 for a recap of the mechanisms). Aquaculture facilities report that nephrocalcinosis often occurs when salmonids held in these conditions are moved to new tanks for grow-out (Sommerset et al., 2022). If water PCO_2_ and PO_2_ are lower in the new tanks, then PCO_2_ in the blood plasma and primary urine will quickly fall while [HCO_3_^−^] in the plasma and primary urine will stay high for some time (e.g., Hobe et al., 1984; Wheatly et al., 1984; Perry et al., 1987; Perry et al., 2010). This may be the proximate trigger. The pH will quickly rise in the blood plasma and primary urine during this period, creating optimal conditions for nucleation, precipitation, and aggregation events to occur in urine that still has elevated Ca^2+^, Mg^2+^, NH_4_^+^, HPO_4_^2-^ and HCO_3_^−^ concentrations. As discussed earlier, the kidney stones will form at pHs above 6.8 (carbonate apatite) and 7.2 (struvite), and this process can occur in less than 2 h for the former, somewhat longer for the latter (Olyniszy et al., 2015):

To make matters worse, once stones start to form, this will further increase the pH of the pre-urine, thereby accelerating the rate of aggregation (Olzinsky et al., 2015). Once the stones aggregate locally into larger stones in the nephron tubules, inflammation and cell damage will disrupt multiple transport mechanisms, and create mechanical blockages to flow. Reduced urine flow itself will further accelerate stone formation. Keep in mind that this is still very early in the process, and it may take weeks or months for the damages to be visible through the use of X-ray or macro-scoring (Klykken et al., 2022b). The issues may further continue as producers use more salinity in the water (brackish water; 12–15 ppt), salted feed (even higher in salts than commercial feed) and/or calcium and bicarbonate buffers (only in RAS) which may further increase Ca^2+^, Mg^2+^ and HCO_3_^−^ levels, having a cumulative effect. Long-term pathophysiological disturbances in ion and water regulation, acid-base regulation, hormone balance, immune response and metabolism will occur, impacting overall fish health and welfare. In the future, better understanding of the urine pH in different environmental settings and pH thresholds for aggregation of kidney stones of different composition will be useful. As these aggregation processes likely start very early, disrupting transport mechanisms in the nephron, an important objective will be to identify key biomarkers involved in the different transport pathways that may help us detect disturbances very early, such that adjustments in O_2_ and CO_2_ levels, water chemistry, and feed composition can be done early in development in modern aquaculture.

## Future research directions

### Expression profiling along the nephron

Section-specific profiling of gene and protein expression in all sections of the fish nephron is a promising advance. The detailed localization of the relevant transporters in acid-base, ammonia, phosphate, calcium, and magnesium regulation to the specific segments (proximal I-, proximal II-, distal-, collecting tubule and collecting duct) provides important information. This can be done through either manual separation (Fehsenfeld and Wood, 2018) or laser capture micro-dissection (Madsen et al., 2020). Combining such methods with the application of scanning ion-selective electrode techniques (SIET; Fehsenfeld et al., 2018) and/or electrophysiology (Kurita et al., 2008; Weiner and Verlander, 2011) using various experimental pharmacological inhibitors/activators and pre-treatments should be pursued with a range of teleost species to better understand the function of these different transport pathways. Additionally, single-cell technologies such as RNAseq and transcriptomic profiling of specific nephron regions would be beneficial to better understand transport mechanisms in physiology and during the development of kidney disease (Potter, 2018). These technologies are already being applied in clinical nephropathology in humans where the main drawbacks at this point are high costs per sample and analytical complexity (Stewart and Clatworthy, 2020).

### Integrative studies on the functional roles of the kidneys

Advances in immunochemical and molecular approaches in the past decade have been accompanied by a decline in the use of more traditional physiological methods, approaches that give a more holistic understanding of kidney function in fish at the whole organism level. Only by utilizing catheterization techniques can you collect urine, measure urine flow rate (UFR) and glomerular filtration rate (GFR), whereby the researcher can determine the rates of excretion, secretion, reabsorption, and plasma clearance of different solutes (e.g., acid-base equivalents, ammonia, phosphate, etc.) in fish (Wood and Patrick, 1994). This becomes a very powerful approach when combined with either section-specific mRNA localization and expression (*in situ* hybridization) and/or protein localization (immunohistochemistry and Western blot), and/or SIET analysis of transport rates and/or single-cell technologies, all of which are useful when finding the location of the target solute carrier or channel. These can often also identify whether the transporter is located on the apical or basolateral membrane of the tubule cell. Several of the transporters addressed in the current review are likely located in the same cell(s). Thus, co-localization of transporters/channels is another approach that would deepen our understanding of the co-operative actions these have in dealing with acid-base, ammonia and phosphate regulation. We now know, for example, that Rh proteins, Na^+^ and H^+^ transport pathways, and carbonic anhydrase are organized into a functional metabolon in the freshwater gill (Wright and Wood, 2009; Ito et al., 2013). The same may be true for these and other transport systems in the fish kidneys (Takvam et al., 2021a). In addtion, a better understanding of paracellular transport (tight junctions proteins; claudins) in conjuction to the trancellular transport (adressed in this review) and their role in acid-base balance, which convincingly have been demonstrated to play an important role in the goldfish kidneys (Figure 3; Fehsenfeld et al., 2019). Future studies should include the individual and collective roles of paracellular and trancellular transport pathways in the fish kidneys. In the future these integrative approaches should be combined in order to better understand complex issues in fish. For example, formation of kidney stones (nephrocalcinosis) is a major issue in intensive aquaculture but so far approaches have usually focused on diagnostic tools (histopathology, blood chemistry, X-ray analyses e.g., Harrison and Richards, 1979; Klykken et al., 2022a; Klykken et al., 2022b; Minarova et al., 2023) rather than on understanding the underlying molecular and physiological mechanisms or the consequences of the disease for normal renal function. While such diagnostic approaches can provide very useful tools to detect the disease, they bring us no closer to finding the root cause of the problem, which requires more integrative understanding on how these transport systems work in the kidneys. The collection of extensive water chemistry and feed composition data in combination with this integrative understanding would be valuable towards solving the problem.

### Comparative studies on mammals and teleosts

Research on mammalian models is more extensive and often better funded due to the many underlying pathological conditions of renal physiology in humans. Fish researchers can learn a great deal from following the mammalian kidney literature as highlighted in several previous sections in this review. However, benefits may also go in the opposite direction: the ability of euryhaline fishes to rapidly adapt to very different environments, and the plasticity of their kidneys where drastic changes in filtration and flow rates (GFR and UFR) as well as substantial shifts (upregulation, downregulation, and net reversal of direction) in their transport systems may provide a powerful experimental model. For example, human kidney disease is often related to reductions in glomerular filtration rates and changes in transport mechanisms. Normal teleost kidney function in freshwater (high GFRs and UFRs) may be a proxy for normal function of the human kidneys, whereas normal teleost kidney function in seawater (low GFRs and UFRs) may be a proxy for abnormal function of the human kidneys. The ability of many teleost fishes to acclimate to high ammonia, hypercapnic, hyperoxic, hypoxic, acidic, and alkaline environments is remarkable, and renal acid-base regulatory function plays a key role in these adjustments. Human diseases ranging from obstructed breathing to liver failure and diabetic coma present some of these same physiological impacts on the human kidneys. Indeed, most of the protein domains related to kidney diseases in humans are present in fish which is why the zebrafish is one of the most popular experimental models for research for human disease (e.g., Bradford et al., 2017). Collectively, increasing our knowledge on the kidneys both at a molecular and physiological levels will be beneficial in addressing these issues in the future as kidney disease is a large and ongoing problem in both mammals and fish.
